# Polyphenol-Rich Extract from Propolis Reduces the Expression and Activity of* Streptococcus mutans* Glucosyltransferases at Subinhibitory Concentrations

**DOI:** 10.1155/2016/4302706

**Published:** 2016-03-23

**Authors:** Jorge Jesús Veloz, Nicolás Saavedra, Marysol Alvear, Tomás Zambrano, Leticia Barrientos, Luis A. Salazar

**Affiliations:** ^1^Center of Molecular Biology and Pharmacogenetics, Scientific and Technological Bioresource Nucleus (BIOREN), Universidad de La Frontera, Avenida Francisco Salazar 01145, 4811230 Temuco, Chile; ^2^Departamento de Ciencias Biológicas y Químicas, Facultad de Ciencias, Universidad San Sebastián, Campus Los Leones, Lota 2465, 7510157 Providencia, Santiago, Chile; ^3^Departamento de Ciencias Químicas y Recursos Naturales, Facultad de Ingeniería y Ciencias, Universidad de La Frontera, Avenida Francisco Salazar, 01145 Temuco, Chile

## Abstract

Tooth decay is an infectious disease, whose main causative agent identified is* Streptococcus mutans* (*S. mutans*). Diverse treatments have been used to eradicate this microorganism, including propolis. To date, it has been shown that polyphenols from Chilean propolis inhibit* S. mutans* growth and biofilm formation. However, the molecular mechanisms underlying this process are unclear. In the present study, we assessed the effect of Chilean propolis on the expression and activity of the glycosyltransferases enzymes and their related genes. Polyphenol-rich extract from propolis inhibited gene expression of glycosyltransferases (GtfB, GtfC, and GtfD) and their related regulatory genes, for example,* VicK*,* VicR*, and* CcpA*. Moreover, the treatment inhibited glucosyltransferases activity measured by the formation of sucrose-derived glucans. Additionally, an inhibitory effect was observed in the expression of SpaP involved in sucrose-independent virulence of* S. mutans*. In summary, our results suggest that Chilean propolis has a dose-dependent effect on the inhibition of genes involved in* S. mutans* virulence and adherence through the inhibition of glucosyltransferases, showing an anticariogenic potential of polyphenols from propolis beyond* S. mutans* growth inhibition.

## 1. Introduction

*Streptococcus mutans* (*S. mutans*) is considered to be the principal causative agent of human dental caries among a wide variety of microorganisms detected in the oral cavity [1, 2]. Although the etiology and factors related to dental caries development are well known, this pathology remains a public health issue due to its increased prevalence in recent reports [3]. Therefore, the search for new therapeutic strategies holds great interest. Polyphenols from different botanical sources have emerged as promissory therapeutic agents due to their wide range of biological activities [4]. Propolis is a resinous substance collected by bees (*Apis mellifera*) from different vegetable species [5]. Several pharmacological properties have been described for the extracts of propolis, such as antidiabetogenic, antiatherogenic, antimicrobial, and antifungal properties, which are related mainly to the polyphenols content of propolis samples [6–9]. Since chemical studies have determined a relationship between propolis composition and the region from where propolis is collected together with the plant source of each area [10, 11], the biological activities of propolis can vary depending on the same conditions aforementioned. We have previously reported antimicrobial activity of Chilean propolis for* S. mutans* [7] and the inhibition of biofilm formation by these microorganisms [12]. However, the underlying molecular mechanism involved in this inhibitory effect by Chilean propolis remains unclear.

The capacity of* S. mutans* to form a biofilm on the teeth surface is considered to be the most important virulence mechanism related to its cariogenicity, which involves sucrose-dependent and sucrose-independent processes. Glucosyltransferases (Gtfs) are involved in the sucrose-dependent mechanism, allowing* S. mutans* the synthesis of both water-soluble and water-insoluble glucans from sucrose [13, 14]. These sucrose-derived glucans favor bacterial adhesion to the tooth enamel by binding to hydroxyapatite minerals (HA) and enabling the interaction between microorganisms. Gtfs from* S. mutans* differ on the types of glucans they can synthetize and the roles performed in biofilm formation. GtfB synthetizes insoluble glucans with *α*-1,3-linkages that facilitates cell aggregation in stable biofilms by mediating the interaction between* S. mutans* bacteria. In contrast, GtfD forms mainly soluble glucans with *α*-1,6-linkages that contain an hydrophobic domain, allowing the interaction with salivary proteins in the pellicle. GtfC produces both soluble and insoluble glucans showing the highest affinity for HA among other Gtfs [15]. The expression of Gtfs is influenced by environmental factors of the oral cavity, for example, pH and carbohydrate availability, but it is also affected by the expression of other regulatory genes such as* vicR*/*vicK*, which belongs to the* vicRKX* operon in the* S. mutans* chromosome [16], and the catabolite control protein A (CcpA) [17]. Regarding the sucrose-independent mechanism, it is less important than sucrose-dependent mechanisms, but it also participates in the primary adherence process in biofilm development, involving the interaction between salivary agglutinins and the surface protein adhesin SpaP (I/II antigen) on the* S. mutans* bacterial wall, which is encoded by the* SpaP* gene [18, 19].

The present study aimed to evaluate the effect of subbactericide concentrations of polyphenol-rich extract from Chilean propolis on the expression of glucosyltransferases and regulatory genes as a possible mechanism underlying the propolis inhibitory effect on* S. mutans*.

## 2. Materials and Methods

### 2.1. Polyphenol-Rich Extract from Propolis

Propolis was collected in spring of 2008 from La Araucanía, Chile. The sample was crushed in cold, 30 grams was dissolved in 100 mL of ethanol (70%) and mixed in constant agitation for 7 days at room temperature. Subsequently, polyphenol-rich extract of propolis (PEP) was filtered with Whatman 2.0 and centrifuged at 327 g. for 20 minutes at 4°C. Later the solvent evaporated in 60°C for 2 hours in a Rotavapor evaporator (Büchi, R-210, Germany) and dissolved for 24 h with sterile DMSO to obtain 50% w/w extract concentrate propolis extract (EP).

### 2.2. Determination of Total Phenolic Content

To quantify the total phenolic content of polyphenol-rich extract from propolis, we used the colorimetric Folin-Ciocalteu assay. Briefly, 100 *μ*L of sample (PEP or calibrator solution) was mixed with 100 *μ*L of distilled water and 2 mL of Folin-Ciocalteu reagent (Merck, Germany). The resultant solution was gently mixed and incubated during 8 minutes. Finally, 3 mL of sodium carbonate 20% (w/v) was added in a 25 mL flask and incubated for 2 hours. The absorbance of the solution was at measured 760 nm in a microplate reader. For the calibration curve, we used standard solutions of pinocembrin-galangin in a proportion of 2 : 1 [11]. Thus, the results of quantification are expressed in equivalents of pinocembrin-galangin mixture (*μ*g mL^−1^).

### 2.3. *Streptococcus mutans* Isolation and Culture Conditions

*Streptococcus mutans* strains were obtained from salivary samples of children with active tooth decay. The samples were cultured in petri plates with Columbia agar enriched with sucrose (1%) in an anaerobic container (Becton, Dickinson and Company, NY, USA). Then, culture plates were incubated at 37°C and 5% of CO_2_ for 24 hours. Finally,* S. mutans* was identified using polymerase chain reaction (PCR) as previously described [20].

### 2.4. Quantification of Relative Gene Expression in* S. mutans* Cultures under Polyphenol-Rich Extract Treatment

To evaluate the activity of the PEP on glucosyltransferases and other virulence-related genes,* S. mutans* was cultured in trypticase soy broth and treatments were applied using a macrodilution sensitivity test scheme with PEP concentrations ranging between 0.1 and 1.6 *μ*g mL^−1^. Moreover, cultures exposed to DMSO and chlorhexidine were used as a vehicle control and positive control, respectively.* S. mutans* cultures were incubated during 24 hours in the conditions above described [21]. Total RNA was isolated from cultures using TRIzol reagent (Ambion, USA) according to manufacturer's instructions. Then, cDNA was synthesized using a reverse transcriptase reaction starting from 1 *μ*g of total RNA and using 200 ng of random primers and 200 U of RevertAid M-MuLV Reverse Transcriptase (Promega, Madison, WI, USA) in a final volume of 20 *μ*L. Glucosyltransferases B, C, and D and* VicK*,* VicR*,* SpaP*, and* CcpA* genes were amplified using real time PCR in a StepOne Real Time PCR System (Life Technologies, USA).

For real time PCR amplification, we used 0.5 *μ*L of each forward and reverse primers (200 nM); 12.5 *μ*L of Fast SYBR® Green Master Mix (Life Technologies, USA); 1 *μ*L of cDNA sample and RNAse-free water up to complete 25 *μ*L of final volume. The amplification program was performed in the following conditions: initial denaturation at 95°C for 10 minutes and 40 cycles of 15 seconds at 95°C, with an extension stage at 60°C for 1 minute. Results were analyzed by the comparative cq method (2^−ΔΔCq^) [22] using the 16S rRNA of* S. mutans* as reference gene. All these experiments were completed by triplicate and results were expressed as relative expression in arbitrary units. The sequence of primers used for this analysis was: GtfB: 5′-AGC AAT GCA GCC AAT CTA CAA AT-3′ and 5′-ACG AAC TTT GCC GTT ATT GTC A-3′; GtfC: 5′-CTC AAC CAA CCG CCA CTG TT-3′ and 5′-GGT TTA ACG TCA AAA TTA GCT GTA TTA-3′; GtfD: 5′-CTT TGG TTC AGA CGG TGT TG-3′ and 5′-CTG CTT TTG ACT TGT TTT CCG-3′; SpaP: 5′-CAG TAC CTG ACT TGA TAA TAA CAC C-3′ and 5′-TCC CTG CAA GAA TCA CTC AGA A-3′; VicR: 5′-CGC AGT GGC TGA GGA AAA TG-3′ and 5′-ACC TGT GTG TGT CGC TAA GTG ATG-3′; VicK: 5′-CAC TTT ACG CAT TCG TTT TGC C-3′ and 5′-CGT TCT TCT TTT TCC TGT TCG GTC-3′; CcpA: 5′-CCG TGA AGC GGG AGT GTC CA-3′ and 5′-TGC CAA ACC ACG CGC CAC AG-3′; 16S rRNA: 5′-TGG AAA CGA TAG CTA ATA CCG CAT A-3′ and 5′-TAA TAC AAC GCA GGT CCA TCT ACT A-3′.

### 2.5. Estimation of Glucosyltransferases Enzymatic Activity

To obtain a crude extract of glucosyltransferases from cultures, the cells were suspended in a potassium phosphate buffer (20 mM) at pH 6.8 and then centrifuged at 10 000 g at 4°C. The enzymes were precipitated with 80% sulfate of ammonium solution. The pellet was resuspended in 500 *μ*L of buffer potassium phosphate for later ultrafiltration in a 10 KDa and 3000 MWCO Vivaspin500 (GE Healthcare Life Science, USA). Finally, a phenylmethanesulfonyl fluoride solution PMSF (1 mM) and sodium azide at concentration of 0.02% were added [23]. For the enzymatic reaction, 50 *μ*L of crude glucosyltransferases extract and 50 *μ*L of sterile sucrose (0.1 M) in potassium phosphate buffer containing PEP in concentrations ranging from 0.1 to 1.6 *μ*g mL^−1^ were added. These solutions were incubated at 37°C for 6 hours in 96-well microplates. After incubation, the content of each well was transferred to a microtube and centrifuged at 10 000 g during 10 minutes. For estimation of water-soluble glucans, the centrifugated liquid was transferred into a microplate and mixed with 10 *μ*L of concentrate sulfuric acid and 10 *μ*L of phenol. Finally, the microplates were incubated at 95°C and the absorbance was quantified at 490 nm in a microplate reader. For water-insoluble glucans assessment, the pellet obtained after centrifugation was dissolved in 300 *μ*L of sodium hydroxide (1 M) and the absorbance was quantified in the same conditions [24]. These assays were performed by triplicate and results were expressed as means ± standard deviation.

### 2.6. Statistical Analysis

Statistical analyses were performed using computational software package Prism 5 (Graph Pad Software Inc., San Diego, USA). The values for comparison of multiple means of individual experiments were estimated by statistical analysis of variance (ANOVA). Significant differences were considered at *p* < 0.05.

## 3. Results

### 3.1. Total Polyphenols Content of Polyphenol-Rich Extract from Propolis

The total phenolics content in PEP was quantified in equivalence of pinocembrin-galangin mixture by the Folin-Ciocalteu reaction to obtain the starting concentration and perform appropriate treatment dilutions for gene expression and glucosyltransferases assays. Thus, PEP solution was determined to contain a concentration of 137753 *μ*g mL^−1^ of total polyphenols.

### 3.2. Effect of Polyphenol-Rich Extract from Propolis on Relative Gene Expression in* S. mutans* Cultures

**Figure 1:**
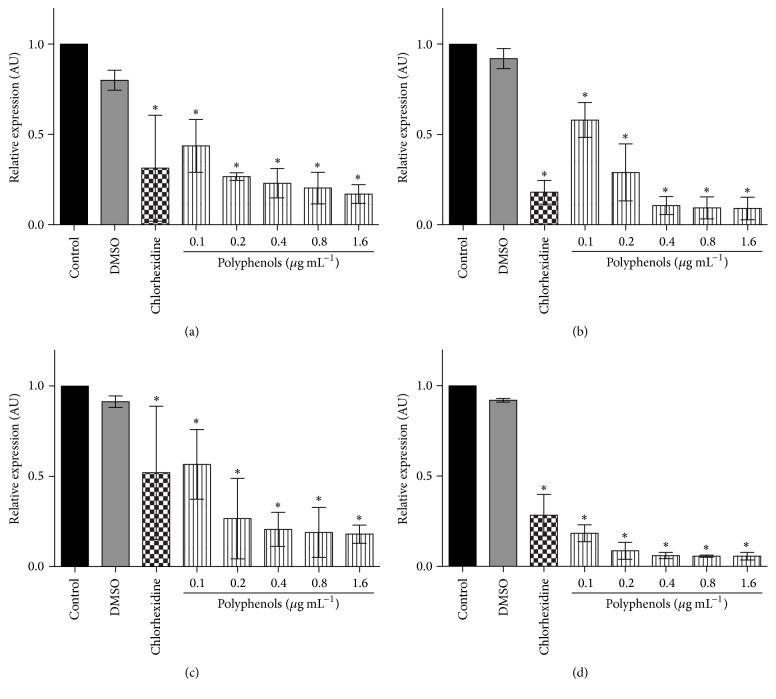
Effect of polyphenol-rich extract from propolis on the expression of genes related with* S. mutans* virulence. (a) GtfB; (b) GtfC; (c) GtfD; (d) SpaP. Statistical significance was determined by ANOVA and Dunnett's multiple comparison test using nontreated cells as control. ^*∗*^ *p* < 0.05.

**Figure 2:**
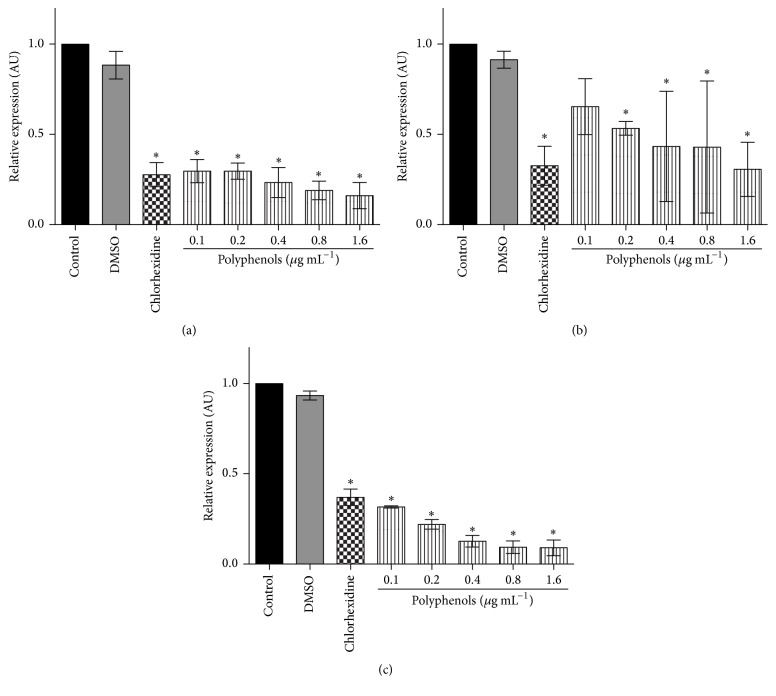
Effect of polyphenol-rich extract from propolis on the expression of regulatory genes involved in* S. mutans* virulence mechanisms. (a)* VicK*; (b)* VicR*; (c)* CcpA*. Statistical significance was determined by ANOVA and Dunnett's multiple comparison test using nontreated cells as control. ^*∗*^ *p* < 0.05.

To evaluate the effect of treatment with polyphenols on gene expression of glucosyltransferases and regulatory genes, cultured* S. mutans* was treated using four noninhibitory concentrations of PEP (0.1, 0.2, 0.4, and 0.8 *μ*g mL^−1^) and one bactericide concentration (1.6 *μ*g mL^−1^) as death control. Values were selected considering the minimum inhibitory and bactericide concentrations obtained for the same extract [12]. Moreover, chlorhexidine was used as positive control due to its well-known bactericide effect on* S. mutans* strains. As expected, chlorhexidine and bactericide concentration of PEP showed a significant reduction on glucosyltransferases or regulatory genes expression, and no effect of vehicle was observed on the expression of all evaluated genes. Moreover, all remaining concentrations of PEP used as treatments inhibited the expression of GtfB, GtfC, GtfD, and SpaP (Figure 1). Similarly, the expression of regulatory genes* VicK* and* CcpA* was reduced in* S. mutans* under PEP treatment at all tested concentrations. However, that inhibitory effect was significant only from 0.2 *μ*g mL^−1^ for VicR gene (Figure 2).

### 3.3. Effect of Polyphenol-Rich Extract from Propolis on Formation of Water-Soluble and Water-Insoluble Glucans by* S. mutans* Cultures

**Figure 3:**
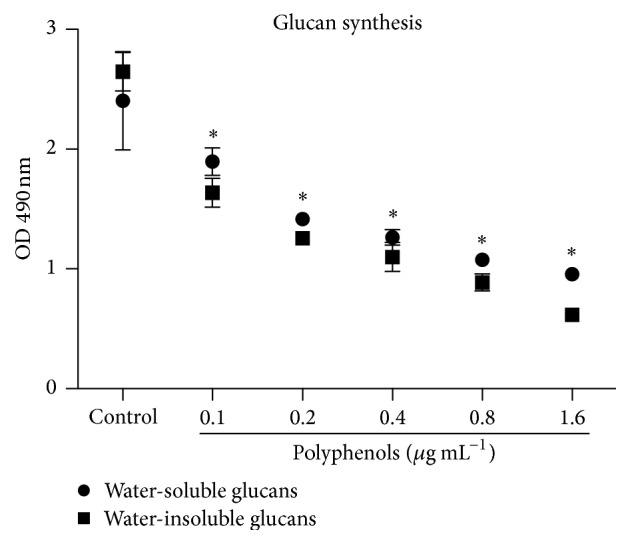
Effect of polyphenol-rich extract from propolis on the metabolic activity of glucosyltransferases. Statistical significance was determined by ANOVA and Dunnett's multiple comparison test using nontreated cells as control. ^*∗*^ *p* < 0.05.

The formation of water-soluble and water-insoluble glucans by* S. mutans* under PEP treatment was assessed in supernatant and pellet of treated cultures. In both cases, glucans formation was significantly reduced at all tested concentrations compared with vehicle-treated cells used as control (Figure 3).

## 4. Discussion

Different studies have been conducted evaluating the effect of polyphenolic compounds with the ability to inhibit* Streptococcus mutans* growth to describe novel therapeutic alternatives for dental caries treatment. These polyphenols have been obtained from several botanical resources including plants [25], fruits [26], green tea [27], and propolis [28, 29]. The antibacterial effect of Chilean propolis has been previously determined, showing minimal inhibitory concentrations (MIC) and minimal bactericide concentrations (MBC) of about 0.9 and 1.3 *μ*g mL^−1^, respectively. Moreover, it was reported that polyphenols from propolis inhibited biofilm formation by* S. mutans *when using subinhibitory concentrations, which probably involves the regulation of physiological mechanisms related to* S. mutans* virulence [12]. In the present study, we evaluated the effect of subinhibitory concentrations of a polyphenol-rich extract from propolis, previously used to inhibit biofilm growth and formation by* S. mutans*, on the expression of virulence genes and glucan formation by Gtfs enzymes. The most important virulence factor of* S. mutans* is its ability to accumulate on dental plaque by formation of biofilm, which is mediated by three distinct Gtfs (GtfB, GtfC, and GtfD) acting by extracellular water-soluble and water-insoluble glucans synthesis from diet sucrose. Teeth colonization occurs by using different pathways, in which the bacteria can generate an attachment on the tooth surface by the antigenic protein SpaP (cPAc or antigen I/II) together with the development of acidic conditions in the oral cavity through Gtfs activities by glucan synthesis, allowing mineral tooth dissolution and cellular aggregation in biofilm structure [30, 31]. After exposition to polyphenols, gene expression of glucosyltransferases showed different levels of repression depending on the concentration of polyphenols tested. The expression of genes encoding GtfB and GtfC showed a stronger inhibition in our model, being repressed more than 40% and 60%, respectively, at concentrations between 0.1 and up to 0.8 *μ*g mL^−1^, with a dose-dependent effect. Similarly,* S. mutans *cultures exposed to polyphenols showed that GtfD was inhibited in approximately 40% or more at subinhibitory concentrations starting from 0.2 *μ*g mL^−1^. Since these Gtfs are decisive in biofilm formation through the synthesis of insoluble glucans in virulent phenotypes, its inhibition can result in reduced biofilm formation, which is consistent with what is previously published for Chilean propolis treatment [12, 32, 33]. Gtfs are regulated at the transcriptional level in response to environmental conditions such as pH, carbohydrate availability, and cell density. Moreover*, S. mutans* displays open reading frames (Orf) for Gtfs regulation; these regulatory factors are associated with two-component regulatory systems (TCSs) as VicK/VicR [34, 35]. Concordantly, PEP treatment results in the inhibition of* VicK* and* VicR* expression; however, the effect was not dose-dependent, suggesting that Gtfs inhibition might involve other regulatory mechanisms. VicK, that corresponds to a membrane-bound sensor histidine kinase (HK), showed a highly decreased expression in approximately more than 50% compared to control in gene expression quantification assays. HKs components are bifunctional proteins having both kinase and phosphatase activities. VicR belongs to the OmpR family of RRs; it is conformed by a helix-turn-helix DNA binding motif and it is involved upstream in virulence genes, including* GtfB*,* GtfC*, and* GtfD*. The biochemistry of the VicR/VicK TCS is important to understand antimicrobial resistance mechanisms and for the development of new potential therapies [36]. Carbon Catabolite Repression (CCR) genes such as CcpA can also regulate* S. mutans* metabolism and adherence on tooth colonization and biofilm formation, integrating complex regulatory networks that activate or silence some genes in response to carbon source and availability [37]. Gene expression of* S. mutans* showed important* CcpA* inhibition following PEP treatment, reaching under 30% of expression in relation to control cells, at low concentrations of PEP in a dose-dependent manner. CcpA is critical for* gtfB *and* gtfC *expression, because CcpA inactivation results in a marked decrease in the levels of Gtfs promoter activity [38], suggesting that inhibitory dose-dependent effect of PEP on Gtfs expression might be triggered by its repression. Moreover, enzymatic activity of Gtfs estimated by insoluble and soluble glucans formation was also affected by PEP at subinhibitory concentrations, confirming a functional effect of Gtfs inhibition. SpaP expression was also reduced by PEP treatment, contributing to biofilm inhibition by a sucrose-independent mechanism, and may lead to the anticariogenic action of polyphenols [18, 19].

The polyphenolic compounds contained in Chilean propolis are diverse and include a variety of flavonoids and phenolic acids [12]. Among the principal components found within Chilean propolis, pinocembrin stands out for its high content [7]. This compound has been identified as a promising pharmacological candidate, mainly for being described with numerous biological activities including antimicrobial, anti-inflammatory, antioxidant, and anticancer effects [39]. Since the present study did not include the evaluation of individual polyphenols, additional studies are required to identify the compound responsible of the modulatory effects shown by PEP treatment.

## 5. Conclusion

In summary, our results suggest that Chilean propolis has a dose-dependent effect on the inhibition of genes involved in* S. mutans* virulence and adherence through the inhibition of glucosyltransferases, showing an anticariogenic potential of polyphenols from propolis beyond* S. mutans* growth inhibition.
